# Environmentally Friendly Approach to the Reduction of Microplastics during Domestic Washing: Prospects for Machine Vision in Microplastics Reduction

**DOI:** 10.3390/toxics11070575

**Published:** 2023-06-30

**Authors:** Aravin Prince Periyasamy

**Affiliations:** 1 Textile and Nonwoven Materials, VTT Technical Research Centre of Finland Ltd., P.O. Box 1000, 02044 Espoo, Finland; aravin.periyasamy@vtt.fi; 2 School of Chemical Engineering, Aalto University, 02150 Espoo, Finland

**Keywords:** microplastics, microfibers, chemical finishing, mechanical finishing, machine-vision, data sharing, supply chain, textile, sustainability

## Abstract

The increase in the global population is directly responsible for the acceleration in the production as well as the consumption of textile products. The use of textiles and garment materials is one of the primary reasons for the microfibers generation and it is anticipated to grow increasingly. Textile microfibers have been found in marine sediments and organisms, posing a real threat to the environment as it is invisible pollution caused by the textile industry. To protect against the damaging effects that microplastics can have, the formulation of mitigation strategies is urgently required. Therefore, the primary focus of this review manuscript is on finding an environmentally friendly long-term solution to the problem of microfiber emissions caused by the domestic washing process, as well as gaining an understanding of the various properties of textiles and how they influence this problem. In addition, it discussed the effect that mechanical and chemical finishes have on microfiber emissions and identified research gaps in order to direct future research objectives in the area of chemical finishing processes. In addition to that, it included a variety of preventative and minimizing strategies for reduction. Last but not least, an emphasis was placed on the potential and foreseeable applications of machine vision (i.e., quantification, data storage, and data sharing) to reduce the amount of microfibers emitted by residential washing machines.

## 1. Introduction

Yarsley and Couzens extolled the benefits of a new class of materials to the world in their book ‘Plastics’ in 1945, at the start of what they called the “plastic age” [1]. They imagined a world where man could make anything he wanted and needed from synthetic materials derived from widely available substances [1]. 1930 marked the beginning of the plastics industry’s ascent to prominence, followed by an extended period of rapid expansion [2,3]. During the 1930s and the early 1950s, various synthetic polymers such as polystyrene (PS), poly(vinyl chloride) (PVC), polyethylene (PE), poly(methyl methacrylate) (PMMA), polyamide (PA), polytetrafluoroethylene (PTFE), polyethylene terephthalate (PET) and polyacrylonitrile (PAN) were developed. However, PET, PP, PE, PA, and PAN were among the major materials utilized in the garments. 

**Figure 1:**
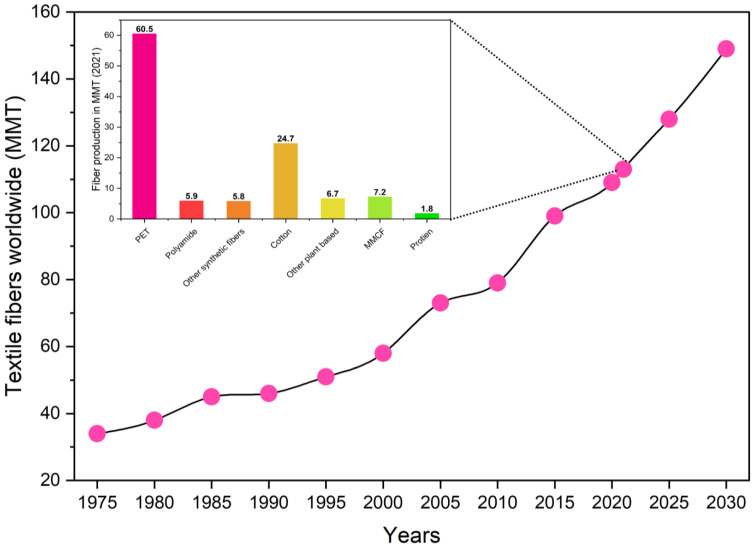
Textile fiber production worldwide from 1975 to 2020, with forecasts for 2025–2030 (in million metric tons); inside shows the different fiber productions [5].

In the past 20 years, the global production of fiber (both synthetic and natural) has expanded from 57 million metric tons (MMT) to 113 MMT in the year 2021, and it is anticipated that the production would reach 145 MMT in the year 2030 [4,5], the textile fiber production worldwide from 1975 to 2020, with forecasts for 2025 and 2030 is shown in Figure 1. As a direct consequence, the average amount of fiber produced per person on a global scale increased from 8.4 kg in 1975 to 14.3 kg in 2021 [5]. Since 1995, synthetic fibers have dominated the textile business, surpassing cotton as the major fiber type, and the worldwide production rate is expected to exceed 65 percent by 2020 [5,6]. Natural and synthetic fibers are utilized in a variety of applications such as garments, household textiles, filtration, and protective clothing. Geographically, China and India are the major manufacturers of synthetic fibers, accounting for 66% and 8% of total production, respectively [7]. 

Microplastics are referred to as plastic particles with a micro size that are typically less than 5 mm in size and have gained importance in recent years due to their abundance (about 8 million tons discharged into the ocean each year) [8,9]. These tiny plastic particles can be found in a wide range of environments, including sediments, beaches, oceans, rivers, lakes, soil, and even in the air [10,11]. Microplastic pollution in the environment has two sources namely primary and secondary. Road markings, tyre wear and tear, marine coatings, plastic pellets, beads, nurdles, and cosmetics/personal care products are examples of primary microplastics. Secondary microplastics are formed as a result of macroplastic degradation during weathering/aging processes [12]. The term “microfibers” is a commonly used phrase within the textile industry, denoting fibers that possess a diameter ranging from 10 to 30 μm [13]. Throughout this article, the focus will be on microfibers that are produced as a result of the domestic washing process. These microfibers are typically of a small size, measuring less than 5 mm.

**Figure 2:**
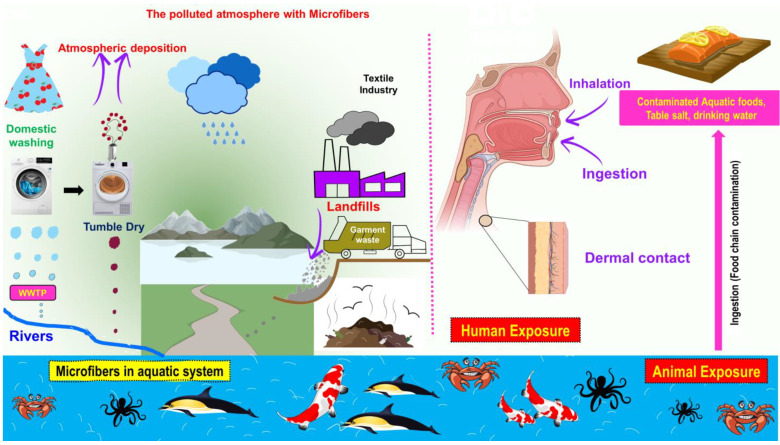
Principal sources of fibrous microplastics and their routes to the human body (i.e., Inside the cartoons are copied from pixabay.com under CC0 license).

Out of the sources described above, synthetic garments are responsible for 35% of the release of microfibers, primarily during domestic washing [14]. In 2004, Thompson and their team found microplastics of a fibrous shape with a diameter of 20 μm in a variety of samples collected from beaches and from estuarine and subtidal sediments in the United Kingdom, as well as in plankton samples collected regularly along routes between the United Kingdom and Iceland since the 1960s [15]. They determined that synthetic polymers such as PAN, PP, PET, and PA existed [16]. Microfibers from textiles and garments can enter the marine environment via a variety of sources, as depicted in Figure 2. 

Nonetheless, in 2011, the Browne et al. [17] study was the first to clearly show how washing our garments could be responsible for marine microplastic pollution. In the textile industry, the sources of microfibers include not only garments but also non-clothing applications such as mattresses, and insulation materials [18,19]. Inside the context of environmental degradation, the textile sector is not a newcomer by any stretch of the imagination. In point of fact, the textile industry is considered one of the primary polluters on the planet due to the use of potentially hazardous chemicals, the consumption of water and energy, the generation of waste, transportation, and the use of non-biodegradable materials for packaging [20,21,22]. In this review article, we will discuss the various eco-friendly and sustainable techniques that may be put to use in order to minimize the quantity of microfibers released during domestic laundry.

The density of microfibers is the primary factor that decides how they are dispersed in the marine environment. The density of seawater is on average 1.027 gm/cm^3^, but it ranges from roughly 1.020 to 1.029 g/cm^3^ depending on factors such as temperature and salinity [23]. In general, PP is microfibers that float in seawater because their densities are lower than the water. Since, PET, PAN, and PA have a higher density than seawater, they have a tendency to sink to the bottom of the marine environment to some extent (Table 1) [24]. However, the qualities of microfibers, such as crystallinity and density, are not intrinsic characteristic properties; rather, they can easily be altered during the process of weathering and aging.

This review focuses on the sustainable way to reduce microfiber emissions by understanding the various textile properties and their influence. Furthermore, it addressed the effect of mechanical and chemical finishes on microfiber emissions, as well as identifying research gaps to guide future research priorities in chemical finishing techniques. It also covered several preventative and minimization approaches for reduction. Finally, the prospective and future applications of machine vision (i.e., quantification, data storage, and data sharing) to minimize microfiber emissions from domestic washing were highlighted.

## 2. Sustainable Way to Reduce the Microfibers Generations

### 2.1. Textile Properties and Their Influence on the Reduction of Microfibers

**Figure 3:**
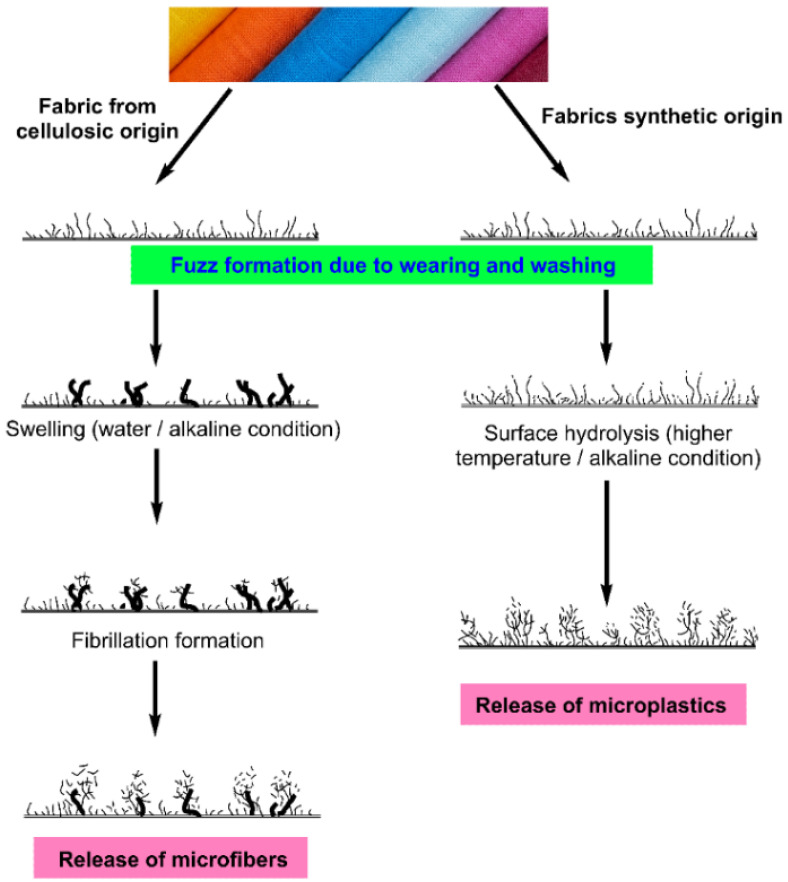
A depiction in the form of a schematic illustrating the process by which microfibers are generated in textiles of cellulosic origin and microfibers are generated in polyester fabrics (Modified and reused from [21] under C.C 4.0 license).

Many factors influence the generation and release of microfibers in synthetic garments during domestic washing, including the type of fabric, fabric geometry (woven, knit, or nonwoven), yarn type (twist, evenness, hairiness, staple fiber, filament, and the number of fibers per cross-section), fabric processing history (i.e., spinning, knitting, or weaving, scouring, bleaching, dyeing, finishing [25], particularly different functional finish) [26,27,28]. The production of microfibers and their subsequent release is predominantly caused by breaking short-staple fibers from the yarn structure [29,30]. Since this staple length of natural fibers can vary, producing evenness in yarn created from these fibers can be difficult. Natural fibers are classified as either short-staple fibers with lengths ranging from 25 to 60 mm or long-staple fibers with lengths greater than 60 mm [31,32,33]. However, because most short-staple fibers are removed during the combing process, combed yarn undoubtedly provides consistency. Staple fibers of the appropriate length are frequently cut from synthetic fibers such as PET, PA, and PAN and regenerated fibers such as Lyocell and Viscose rayon for use in staple yarn manufacturers (~typically 38 mm) [33,34,35,36,37]. In most cases, pilling is produced by the staple fibers in the fabric coming apart at the surface because of the fabric being subjected to mechanical stress. The formation of fuzz is the first step in the pilling process, which can be identified by the gradual separation of fibers from a surface because of the mechanical action of the washing and wearing cycles [38,39]. The fiber frictions, their cross-sectional shape, thickness, stiffness, yarn hairiness, and abrasion resistance all contribute to the formation of fuzz. The protruding fibers (i.e., short fibers) enhanced hairiness in the yarn are responsible for a considerable quantity of microfiber emissions after washing. These microfiber emissions can subsequently cause fuzz (see Figure 3) to form on the surface of the garment. During the process of pill formation, cellulosic fibers such as cotton and lyocell fibers initiate the fuzz formations which are followed to develop the fibrillations [39,40,41,42] and synthetic fibers such as polyester offer a way that is functionally equivalent to one another. The predicted mechanism for the development of microfibers is represented in Figure 3. According to the research that was presented in the cited article [43], the application of fabric treatments to synthetic clothing while they are being washed enhances the risk that the garments may pill.

The percentage of various components employed in the production of the yarn or fabric (for example, in blend fibers, yarns, and textiles) has a major effect on the formation and release of microfibers during laundry. Synthetic fibers, in general, lack the natural convolution or crimp found as compared to natural fibers such as cotton. Because of the weak fiber-fiber interactions that may occur as a result of the mechanical action of washing and tumble drying, the microfibers may become detached from the larger fibers [44]. The structure of the fabric and the yarn has a significant impact on the generation of microfibers; in this specific example, the fleece and pile fabric structures release a significantly higher number of microfibers than the plain-woven fabrics [44,45], this is due to the fact that the surface of fleece fabric is composed of open loops or fibers that stick out in different directions [46,47]. Another study [48] evaluated the effect that different fiber blend ratios had on the quantity of microfibers that were released, and the findings were as follows: a 100% PET woven blouse had the least amount of microfibers released, followed by a 100% PET knitted T-shirt, a 100% PET knit fabric, and a 50% PET/50% cotton knit hoodie. There was no noticeable difference in microfiber emission between 100% PET knitted fabric and 50/50 PET/cotton knitted sweatshirts, all of which were made from staple yarns [48]. Fabrics made of continuous filament fibers, on the other hand, emit fewer microfibers into the air than their short-staple fibers [49]. Knitted sweatshirts made of 50% PET and 50% cotton were found to produce the greatest microfibers in water (80%) and in the air (97%) [49].

According to De Falco et al. [49]. the microfiber emission rate of 100% PET knitted fabric is lower than that of 100% PET woven fabric. In comparison to knitted fabric, which provides some flexibility during mechanical action, the surface rupture of woven fabric is significantly greater due to its stiff structure. Furthermore, the yarn used to manufacture knitted polyester fabric is less hairy than the yarn used to make woven polyester fabric. Perhaps, other studies suggest that knitted fabrics release more fibers than woven fabrics due to their increased structural compactness [45,50]. In contrast, the yarn used to construct synthetic knitted fabric is typically formed of continuous filaments, as it has fewer short fibers, resulting in fewer opportunities for microfiber release. In contrast, the yarn used to construct synthetic knitted fabric is typically formed of continuous filaments, as it has fewer short fibers, resulting in fewer opportunities for microfiber releases. Microfleece is a type of napped and/or mechanically cut knitted terry fabric that is typically made by combining doubled yarn in the warp and a single yarn in the weft. As a result, more microfibers were released from these microfleeces than conventional single jersey knotted fabrics [45]. The amount of microfibers generated by old clothes is approximately 25% greater than that of new clothes [51]. The mechanical structure of fibers decreases with time due to variables such as sunlight exposure, wear, and cleaning. Sunlight contains a wide spectrum of wavelengths that can cause fabric damage in a variety of ways. When a cloth is exposed to UV, visible, and infrared light, it generates additional heat, accelerates fiber oxidation, and hastens fiber disintegration [52]. In addition to these factors, CO_2_, NO_2_, and NO_3_ gas exposure, perspiration, abrasion, friction during wear, and frequent washings and dryings all contribute to fiber wear and tear [53]. It is possible for the mechanical operations that take place during the washing process to damage the staple fibers and cause them to become detached from the yarn structure [54]. Despite the fact that the emission of microfibers is significantly controlled by parameters including the twist of yarns, breaking strength, elongation, bending stiffness, friction, and the softness of the yarn [46,49,55,56], nevertheless, optimizing these parameters have still possible to reduce the number of microfibers released.

**Figure 4:**
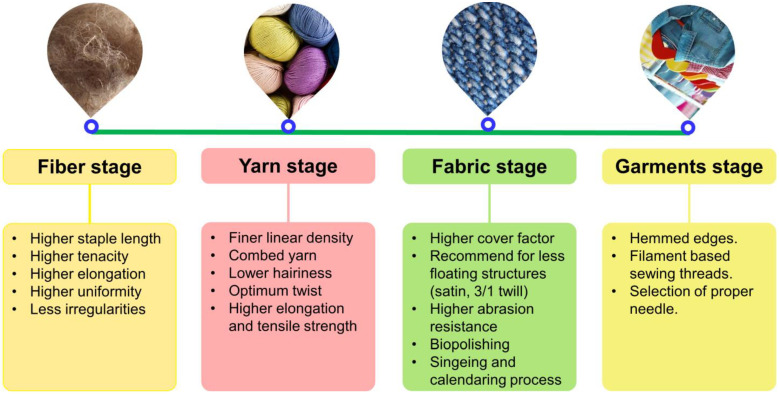
Various textile characteristics are desirable for reducing the number of microfibers and microfibers that are released throughout the washing and drying processes.

A comprehensive description of the textile characteristics and the impact that they have on the minimization of microfibers is presented in Figure 4. Fiber is the raw material for textiles, and the properties of fibers determine the characteristics of textile goods, including how they behave regarding microfiber releases. When considering fibers, choose ones with a longer staple length and continuous filaments that have stronger tenacity and elongation. This helps to reduce the number of microfibers released. The finer the yarn, the greater the elongation compared to the coarser, and it also has fewer short fibers, often known as projecting fibers. As a result, there are fewer opportunities for microfiber release. Fabrics made from combed yarns are preferred for textile production because they have a more uniform length followed by the appearance of carded and open-end spun yarns. Furthermore, the combed yarn contains fewer hairy fibers, which reduces the amount of microfibers emitted.

The higher the twist in the yarn, the greater its strength, which leads to a reduction in the number of generations of microfibers. Because the higher floating structure provides more opportunity for the short fibers in the fabric and yarn structure to become dislodged, a fabric with a higher floating structure, such as satin or 3/1 twill, produces more microfibers than a fabric with a lower floating structure [56]. It is believed that the fabric has a higher cover factor, and its compact structure aids in limiting microfiber emission due to greater fiber-to-fiber contacts. Some of the physical properties of the fabric are inversely proportional to the amount of microfibers released. For example, the stronger the fabric’s abrasion resistance, the fewer microfibers are discharged during routine household washing [49]. Sealing the edges of the garment during preparation offers more possibilities for reducing microfiber emissions. However, friction between the sewing thread and needle operation and the fabric, as well as mechanical damage caused when the needle comes into touch with the fibers, may increase the amount of microfibers released [22].

### 2.2. Washing Parameters and Their Influence on the Reduction of Microfibers

**Figure 5:**
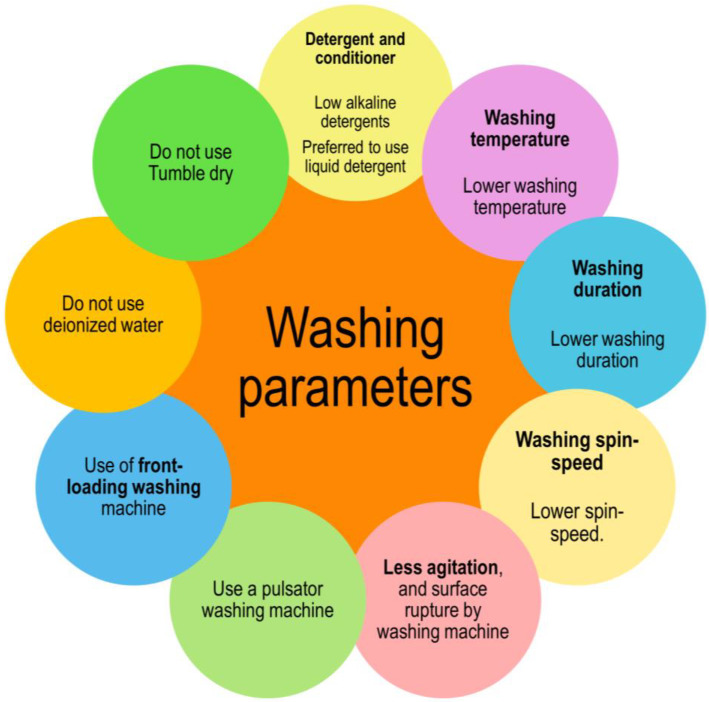
Factors to consider in order to minimize the microfibers/microplastics throughout the laundering and drying processes.

More than 840 million residential washing machines are used globally [57,58]. In such a circumstance, it is critical to optimize the various washing parameters that can help to limit microfiber emission. Several papers on this subject have already been published, which assist in optimizing the various washing parameters [51,59,60,61]. Figure 5 highlights the important parameters that must be considered in order to limit the number of microfibers and microfibers discharged throughout the washing and drying procedures. Due to oxidation and hydrolysis of the fibers formed from polyester, the temperature causes the fibers derived from cellulosic-based materials to degenerate after an extended period of washing [22]. Detergents wet surfaces as temperatures rise, and fibers slip more easily from yarns and fabric structures. It is preferable to utilize liquid detergent formulations based on nonionic detergents rather than formulations based on anionic detergents and inorganic salts. The formulation should show improved washing efficiency at lower temperatures and with higher degrees of water hardness [62]. Increased friction between the clothing and the machine drum, induced by increased washing rotational speed and agitation, results in a greater discharge of microfibers. As a result, the ideal speed reduces the overall microfiber release rate.

In addition to this, the proportion of the garment to the washing liquid that is used in the machine is an important factor in the production of microfibers [63], the authors found that there were 65 mg of microfibers per kilogram of the garment, but that number increased to 125 mg of microfibers when twice as much wash water was utilized. Additionally, the larger water volume results in an increase in the amount of mechanical stress that is placed on the clothing while they are being washed [22,55]. Piol et al. [64] investigated the effectiveness of new additives for washing liquor, such as builders and softeners, in lowering the amount of microfibers released into the environment.

The builders’ primary job is to chelate positive ions (Ca^2+^, mg^+^) from hard water during the washing process. Although anionic surfactants effectively remove grease, oil spots, and stains, it is sensitive to multivalent ions present in hard water. Positive ions bond with the anionic surfactants, generating precipitation on the cloth surface. Furthermore, the Ca ion has the virtue of acting as a glue between the stain and the fabric surface, making it more difficult to remove, and reducing cleaning efficiency. Perhaps more detergents or friction was necessary to achieve the intended cleaning, which is directly proportional to the favoring of microfiber releases.

**Figure 6:**
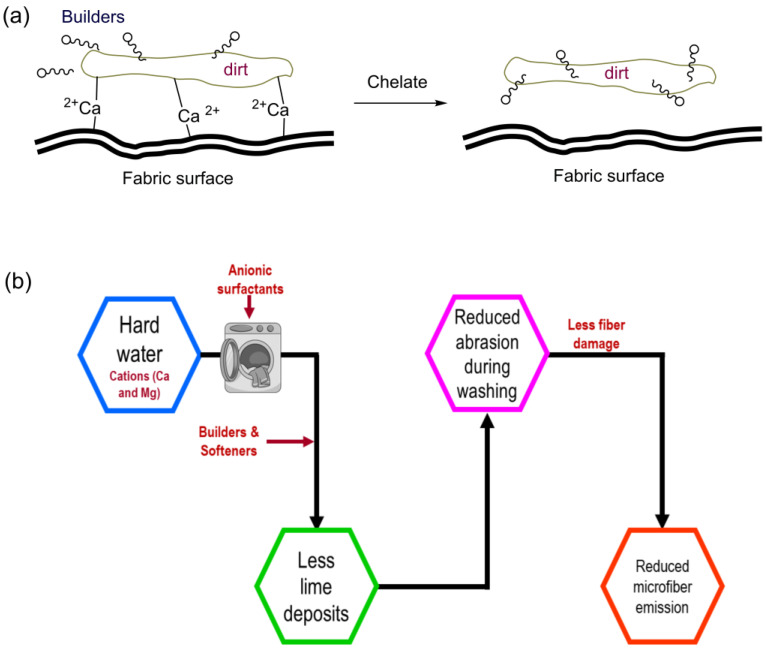
Builders and their impacts on stain removal under hard water (**a**), action on microfiber reduction (**b**).

Nonionic surfactants are easily soluble in water and function well in hard water, although they are less effective in removing soil than anionic surfactants. Nonionic surfactants have the hydrophilic group (i.e., polyoxyethylene group), which offers lubrication to the clothes and may result in a reduction in microfiber release [65], although this is a supposition, and no study effort can substantiate this statement. Figure 6a depicts the chelation reaction on the calcium via the builders on the cloth surface and its influence on stain removal. Furthermore, lime is deposited on fabrics as a result of hard water formation, creating abrasion. As a result, there is severe fiber damage as a result of continuous washings, which generate more microfibers. Builders may be able to decrease all of these effects of hard water by avoiding difficulties (Figure 6b). Builders, on the other hand, deposit, but the amount is minimal during the washing cycle without builders. Enzymes such as cellulases, proteases, lipases, and amylases were added to detergents to improve stain removal and shorten the time it takes to wash laundry [53]. Enzymes such as cellulase facilitate the hydrolysis of -1,4-linkages present in cellulose fibers, leading to the conversion of these fibers into monosaccharides [66]. Consequently, detergents containing cellulase cause the separation of fibers from the surface of the fabric, thereby resulting in a significant reduction in the quantity of microfibers. The uniformity of the cellulase action mechanism renders the processes of biopolishing and cellulase action in detergent indistinguishable. It is imperative to note that the impact of the aforementioned phenomenon is contingent upon the level of concentration.

### 2.3. Mechanical Finishing 

**Figure 7:**
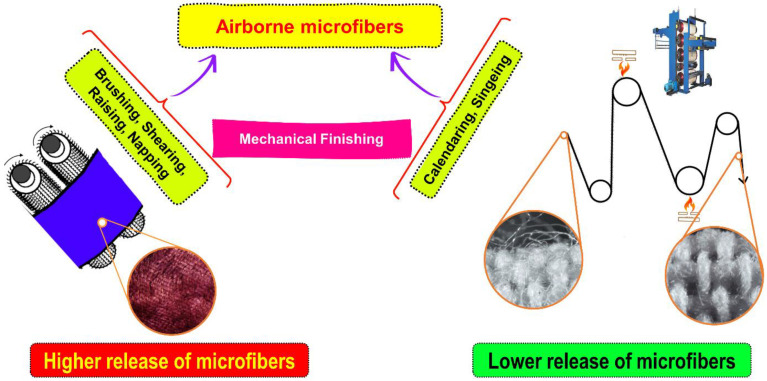
Various mechanical finishing and their impact on microfibers emission during domestic washing.

Mechanical finishes, such as singing and calendaring, help control the dissemination of fiber particles. Singeing is the practice of using a carefully directed open flame to burn away the coarse hairs that have worked their way to the surface of a fabric. Fabric is compressed by passing it between two or more rollers in a process known as calendaring, in which time, temperature, and pressure are carefully controlled [66]. Sanforizing and heat-setting processes are used to shrink and permanently set the woven fabric in both the warp and the weft directions. Mechanical finishes typically offer mediocre longevity and fail to prevent microfiber release after only a few domestic washes. To obtain the fuzz effects on the fabric, the brushing process will take place on the fabric surface with the help of emery rollers offers significant possibilities on the microfiber releases. As the fiber is partially loosened from the yarn’s structure due to the mechanical action of emery rollers. The fabric’s surface structure is significantly changed due to the brushing process, and this modification allows for the freeing of microfibers Figure 7. Increased microfiber release in the first few washes is typically due to trapped microfibers being released. These microfibers were created in the mechanical finishing stage [67]. 

### 2.4. Chemical Finishing

#### 2.4.1. Enzymatic Treatment

A potential solution to the issue of microfiber pollution could be found in modifying the fabrics’ surface levels. Cellulosic clothing undergoes a process called biopolishing, which involves the cellulase enzyme and removes fibrils or microfibrils from the fabric’s surface, offering the reduction of microfiber releases and improving the fabric appearance by reducing the problems such as pilling properties. Using enzymes to break down fibrils results in a cleaner, softer fabric surface [68,69]. The immobilized cellulase has a strong influence on the protruding fibers of the cotton fabric [70]. Visual appearance confirms that the immobilized cellulase provides better removal of fibrils/microfibrils than the cellulase-treated cotton fabric. However, there is little data on how enzymatic treatment’s longevity stacks up against the protrusion of fibers that develops following wash and wear cycles. In another research [71], the issue of microfiber shedding was approached using an eco-friendly method that involved the enzyme lipase and the surface modification of polyester fabric. As a result of the surface modification, the issue of microfiber shedding was mitigated, which led to a reduction in the amount of microfibers that were shed.

#### 2.4.2. Coating and Chemical Finishing

The process of finishing and coating textiles with a range of chemicals, including silicone, polyurethane, and acrylic polymers, may assist to reduce the amount of microfibers that are released into the environment. These polymers form a layer on the surface of the fibers/fabrics, which serves to protect the fibers from abrasion and mechanical strain. Usually, this layer is generated on the surface of the fibers/fabrics. Numerous studies have been carried out in order to investigate the effect of finishing processes on microfibers and to analyze the findings of these studies. Because of its strong attachment to the surface of the textile, the chemical treatment may reduce the number of microfiber releases, which in turn guarantees a reduction in the number of microfibers that are released. In addition to releasing microfibers, these polymeric films also have the potential to release microparticles as a result of the abrasion that takes place while they are being used and washed [72,73]. The different synthetic textiles were coated with a variety of resins, including acrylic resins, polyurethane resins, and silicone emulsions, using techniques such as padding and exhaustion [64]. This trend was observed in both woven and knitted fabrics that were produced out of PET, PP, PAN, and PA. Out of all the materials tested, acrylic resins had the best pilling resistance out of any of the auxiliaries that were chosen. On the other hand, in comparison to the emission of microplastics by other finished fabrics, the emission of microplastics by the acrylic resin-finished fabric is significantly lower. Similarly, the PET fabric that had been treated with silicone emulsion was noticeably softer than the untreated PET fabric, even though it has more possibilities on the pilling formations. The number of microfibers that were released by fabrics treated with silicone emulsion was significantly lesser, as determined by the findings of the laundry tests, in comparison to the number of microfibers that were released by fabrics treated with other chemical finishes. The application of a silicone finish makes the surface of PET fabric softer, which in turn minimizes the amount of friction that takes place during the washing process between the fabric and the detergent, as it is the most important factor contributing to the issue. On the other hand, it was difficult to find any publications that explored the durability of these chemical finishes to evaluate whether or not they are a short-term or long-term remedy for the release of microfibers [21].

#### 2.4.3. Finishing with Pectin

The application of a pectin-based (PEC) finishing to PA fabric demonstrates a considerable reduction in microfiber emission [74]. The two-step process of grafting PEC into the PA surface begins with the synthesis of PEC-glycidyl methacrylate (GMA, 97%) and concludes with the grafting of PEC-GMA onto the fabric (PEC-GMA-PA). Nevertheless, GMA has exhibited toxicity towards the ocular, digestive, respiratory, and dermal systems [75]. The author has selected pectin as it originated from a natural source, which ensures sustainability, and it is cheap, abundant, and can potentially react with fabrics. Maior et al. [76] provided a very clear description of the chemical reaction that takes place between PEC and GMA. The pristine-PA and PEC-GMA-PA fabrics were washed for 45 min at 40 °C with a commercial detergent according to ISO 105-C06:2010. The research discovered that for every kilogram washed, the pristine-PA cloth shed microfibers of 12 ± 222 μm in length, 18 ± 3 μm in mean diameter, and 0.359 kg in total. The PEC-GMA-PA treatment, on the other hand, decreases the microfiber count to 550 ± 384 μm, 16 ± 4 μm μm, and 0.058 g per kilogram of cloth. This amounts to a reduction of microfibers by about 90%.

#### 2.4.4. Finishing with Biodegradable Polymers

The PA fabric was electro-fluidically (EFD) coated with biodegradable polymers, including polylactic acid (PLA) and poly (butylene succinate-co-butyrate adipate) (PBSA) [77]. Treated fabric shows a 90% reduction in microfiber emissions as compared to untreated fabric. The EFD treatment has several benefits, including (a) uniformity of coating, (b) the ability to coat nano-sized particles, and (c) surface finishing of the fabric without compromising its fundamental properties. Microfibers released from washed PLA and PBSA-finished PA cloth are 428 ± 92 and 456 ± 120, respectively. The count of microfibers produced by a single gram of pure PA fabric is around 3966 ± 1425. Additionally, compared to the PEC-GMA finishing [74] on PA fabric, this one reports a 90% reduction in microfibers emissions. EFD technics ensured the uniform coating, and it is distributed across the fabric’s surface during the finishing process, as this layer shields the fabric from the destructive effects of the washing machine’s mechanical agitation and chemical reactions. 

#### 2.4.5. Finishing with Chitosan 

In another study, different concentrations of chitosan were applied to the PET fabric to determine their influence on the release of microfibers [64]. Therefore, 1, 2, and 3% chitosan concentration, respectively, chitosan-finished PET fabric released 1726, 2497, and 2237 microfibers per gram of fabric. On the other hand, the untreated PET fabric released 3047 microfibers. When compared to the sample that had not been treated, the PET fabric that had been treated with chitosan at a concentration of 3% emitted 27 percent fewer microfibers than the untreated PET fabric. The researchers have not presented any findings pertaining to the bonding between chitosan and PET, as well as the fabric’s washing durability and its impact on microplastic generation [64]. On the other hand, there is never a consistent result even when the findings of the tests are repeated on multiple separate occasions. It is possible that this is due to the relatively low affinity of chitosan on the PET fabric. Treatment with this substance is less successful and less long-lasting than treatments involving pectin [74] and PLA/PBSA [77]. 

The findings are encouraging about polymeric chemical finishes. It is possible that the amount of microfibers that are discharged can be reduced if a layer of protection against abrasion is provided by a polymeric film that is covering the fibers. However, to stop additional contamination of the environment and to end the process sustainably, it is very important and necessary to use chemicals and polymers that are not harmful to the environment. However, there are a great many alternatives that have never been examined before and have provided researchers with possible lines of exploration. Some examples of these alternatives include enzyme treatments, plasma treatments, and the use of various biopolymers [78,79].

#### 2.4.6. Finishing on Denim Garments 

**Figure 8:**
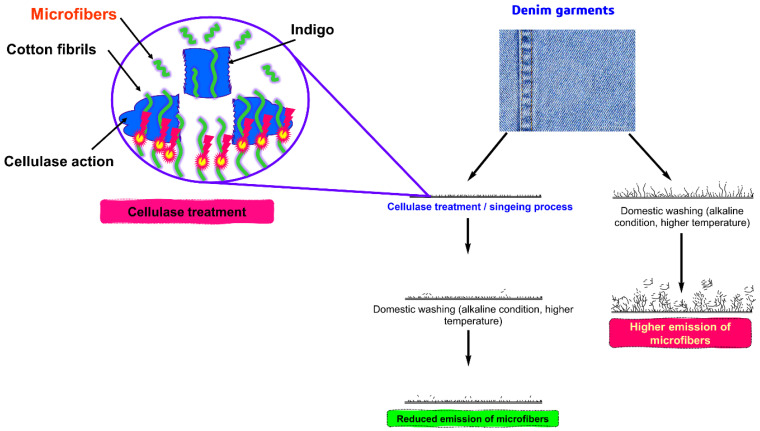
The mechanism for the microfiber generations from denim and their possible finishing techniques to reduce the microfiber generations (Reused from [90], with permission from ACS Creative Commons Permission).

Denim finishing encompasses a wide range of processes that are utilized to achieve the goal of removing the dyes from the surface of the fabric. Stone washing, sandblasting, scraping, whiskers, tacking, grinding, and ripping cuts were some of the techniques that were utilized in these processes [80,81,82,83,84,85,86]. The process of ring dying (i.e., indigo dyes) is used to provide the denim fabric with its signature blue or indigo hue [87,88]. The mechanical fading effect is directly proportional to the amount of abrasion that takes place on the denim surface, as the abrasion process opens up the structure of the yarn and produces an appearance that is soft, and fluffy, each of these finishing processes has enhanced the possibility for reducing the number of microfibers that are removed when the denim is washed in a household washing machine. It is likely that cellulase enzymes are used during the process of removing fuzz filaments from denim during the finishing process [89]. Perhaps, this assertion is not supported by any studies; yet there is hairiness on the surface of the cloth that significantly reduces due to the cellulase treatment which is directly proportional to the reduction of microfibers releases. Figure 8 depicts the mechanism responsible for the formation of microfibers from denim as well as the potential finishing techniques that can be used to reduce the number of microfiber generations.

Table 2 summarizes the potential significant outcomes of several mechanical and chemical finishing procedures on the reduction of fiber fragments in the laundry. 

## 3. Microfibers Prevention and Minimization

**Figure 9:**
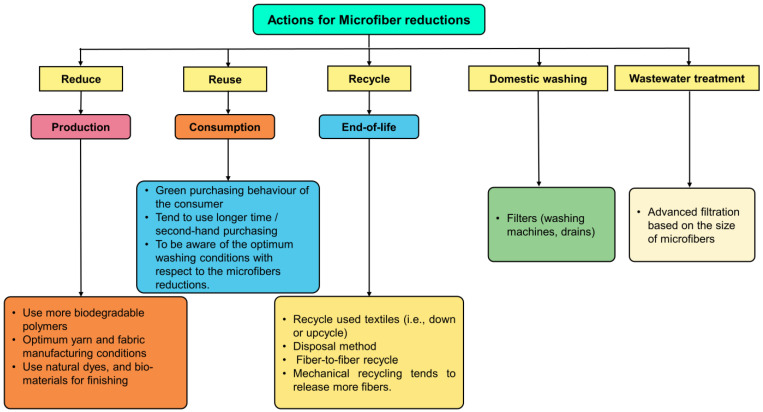
Positive actions toward avoidance and reduction of microfibers and microfiber generations.

Take preventative measures to avoid and restrict the development of microfibers from the very beginning stages of the manufacture of textiles and the usage phase. This is the single most important thing that can be carried out to reduce the amount of pollution that is caused by microfibers and microfibers. Figure 9 presents some of the constructive measures that can be performed to prevent or reduce the number of future generations of microfibers and microfibers.

### 3.1. Production Phase

The global production of synthetic fiber has expanded at an exponential rate over the course of the last 20 years. Consequently, there has been a notable increase in the mean quantity of fiber generated per capita worldwide, with figures rising from 14.0 kg in 2020 to 17.5 kg in 2030 [4,5]. The production of fibers and fabrics that are more durable, enabling longer durations of use and reuse, and that reduce shedding during activities such as wearing, and washing is one potential approach that might be taken to address the problem. Utilizing natural cellulosic materials and developing alternative textile materials, such as those made of bio-based and/or biodegradable polymers, may both be potential alternative solutions for achieving the goal of establishing a bioeconomy that is both completely sustainable and circular. This goal was established by the United Nations in their 2030 Agenda for Sustainable Development. On this occasion, PLA [91], polyhydroxyalkanoate (PHA) [92], polycaprolactone (PCL) [93], polybutylene succinate (PBS) [94], polyhydroxybutyrate (PHB) [95], Ioncell [96], BioCelsol [97], Infinna [98], and Renewcell [99] are the alternatives that are talked about the most frequently. Ioncell, BioCelsol, Infinna, and Renewcell are all examples of what are known as regenerated cellulosic fibers, whereas PLA, PHA, and PHB are all examples of bio-based and biodegradable fibers created from renewable sources. Nevertheless, it appears that not all the offered alternatives are suitable for usage in textiles; furthermore, most of them are not commercialized; and there is no literature on these polymers versus addressing the microfibers issue. It is indeed that there are still a lot of questions about how colored and chemically finished biodegradable plastics can be degraded in different environments. Most studies have been conducted on PLA as a replacement for PET [91,100,101,102]. There are a number of problems that arise when PLA is used in textile manufacturing or applications, such as its low heat resistance, but researchers are hard at work trying to find ways around these issues. Worldwide production of bioplastics increased by 16% in 2021, reaching 2.4 MMT, meanwhile, 1.6 MMT of biodegradable bioplastics were expected to be produced in 2021. Predictions suggest that growth will continue uninterrupted and that by 2026, global bioplastics production might reach 7.6 MMT [103]. However, only a small proportion of these polymers are used in textiles (~10%) [104]. One of the most crucial factors to think about when evaluating the pros and cons of biopolymer use is the production method’s impact on the surrounding ecosystem. Biopolymers have been found to be more environmentally friendly and energy efficient than petroleum-based polymers through life cycle assessments (LCAs) [105,106]. One of the benefits of biodegradable polymers is that they can be disposed of in a variety of ways, including recycling, home and industrial composting, landfilling, and burning. In terms of how it interacts with the environment, the fact that it decomposes naturally is generally viewed as an advantage. Despite the previously described benefits, it is crucial to note that it is not viable to replace all synthetic materials with bioplastic materials. This is especially true when considering the massive manufacturing volume required. Other processes, such as textile finishing, need to be improved in order to reduce their negative effects on the microfiber releases also the quantity of toxic chemicals that are released into the environment as a result of these operations [107]. It is also carrying the prewashing treatment on the garments before they go to the store might influence the reduction of microfiber releases. When transitioning to biodegradable fibers, the industry must take into account two crucial factors. The first pertains to cost, which is currently high and limits the number of viable applications, such as face masks. Typically, the implementation of biodegradable fibers is known to enhance sustainability and mitigate the prevalence of toxic contamination in both marine and terrestrial environments. Nevertheless, the introduction of functional additives, such as dyes and finishing chemicals, has been observed to yield microfiber emissions with diminished biodegradability [20]. The production of fully biodegradable fibers necessitates the incorporation of biodegradable additives, such as colorants derived from natural sources, and other functional finishes. 

### 3.2. Consumption Phase

In 2022, the average person in Europe will have accumulated more than 15 kg of textile waste. The majority of waste produced from textiles is comprised of worn-out clothing and other types of household textiles that are no longer wanted by their owners (i.e., consumers). This accounts for around 85% of the total waste produced [108]. It is possible that a short lifespan for a garment is not necessarily the consequence of the item’s poor quality or lack of durability; rather, it may be linked to the actions of consumers. Important as they are, textiles have the ability to leave a substantial imprint on the environment in terms of the additional resources utilized, the amount of carbon dioxide emissions, the amount of chemical emissions, and the quantity of water that is consumed. Since a significant proportion of microfibers are generated during the initial wash cycles of newly purchased clothing items. Therefore, it is imperative for brands and manufacturers to consider and integrate the prewashing of garments prior to their distribution to retail establishments. Nonetheless, a primary constraint of this procedure is the potential escalation of production expenses. Consequently, it may be beneficial for brands and manufacturers to raise consumer awareness regarding environmentally conscious purchasing practices. Furthermore, it is probable that microfibers can be effectively removed during the manufacturing process rather than at the household level. In addition to that, the educational programs are advocating extended usage by customers in an effort to combat the phenomenon known as fast fashion. These concepts need to be supported by activities within the textile manufacturing industry in order to produce apparel that is of a higher quality and has a longer lifespan. One other thing that has to be carried out is to devise a method that is streamlined for the distribution and selling of pre-owned clothing can help to reduce the virgin sources. 

### 3.3. End of Life, Recycling, and Disposal Phase

It is vital to cut down on the emission of microfibers by increasing the collection of garment waste that has been abandoned and finding new applications for those garments rather than simply tossing them away. This will help reduce the number of microfibers that are released into the environment. In order to extend the usable lifespan of textile goods, used clothing may be repurposed into new things such as industrial rags, furniture decorations, handbags, backpacks, promotional textiles, and so on. Therefore, a matched sequence of activities relevant to the disposal and processing of worn textiles has to be devised as well as made more generally known to the general public. For fiber-to-fiber recycling, recycled PET (i.e., chemical recycling; from monomeric form) added to textiles increases their tensile strength and abrasion resistance, which is an interesting observation [109]. The textile recycling technologies that are now accessible face a significant obstacle in the form of the various blending of materials, coatings, dyes, etc [110]. In order to increase recovery and conversion efficiency and to lessen the effects on the characteristics and quality of the materials, additional development is also needed in the area of the chemical recycling of plastic polymers [111,112,113,114]. However, we cannot overlook the interconnected problems of pollution and energy use. The utilization of enzymatic digestion has been observed as a viable method for the isolation of natural fibers from blended fabrics that are composed of both natural and synthetic polymers. This process is particularly effective for fabrics that consist of cotton, wool, and silk in combination with PET and Polyamide. However, these techniques have not yet been commercialized. Afterward, the residual abrasive PET or Polyamide may be recycled into PET or Polyamide fibers, and then the fibers can be spun into yarn. The European Union, and notably Finland and Sweden, have made significant progress in the development of regenerated cellulose fiber because of the upscaling potential of this technology. To do this, the paper and pulp business may be quickly and simply transitioned into the regenerated cellulose fiber sector. There has been a surge in interest in studying regenerated cellulose fibers, and the market is expected to develop at a CAGR of 10.5% between 2016 and 2026, reaching 7.1 million metric tons by 2021 [5,97,115]. 

### 3.4. Domestic Washing and Wastewater Treatment Phase

One of the most essential steps in the process of reducing the amount of microfibers is the domestic washing and wastewater treatment phase. In general, the optimization of washing techniques to minimize the abrasion of synthetic fabrics can assist to prevent emissions. This can be accomplished by washing the synthetic garment in colder water. The amount of detergent used, the pace at which the washing is conducted, and the volume of water utilized may all be optimized to a lesser extent. 

**Figure 10:**
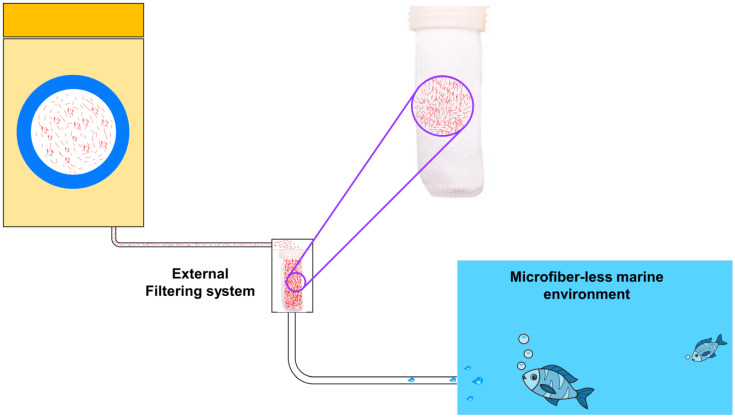
Schematic representation of extra filtering system for washing machine.

Installation of a removable stainless-steel filter at the drain connection point or utilization of an external filtering system at custom pipe connections is both realistic solutions that can be considered as additional preventative measures, respectively [26,116]. It was recently revealed that a filter that had an installed stainless steel filter with a mesh of 150–200 µm could retain 87% of the total number of microfibers that were present in washing effluent [44], the typical filtration system has been explained in Figure 10. France is the first country in the world to have measures in place to decrease the quantity of microfiber pollution that is caused by laundry as a result of the recent adoption of a law that mandates all new washing machines must contain microfiber filters by the year 2025 [117]. There are presently no state or federal rules in the United States that particularly address the problem of microfiber contamination from washing, even though numerous state governments have acknowledged that microfibers are a concern. Nevertheless, a bill is currently being considered in the legislature of the state of California that would establish a pilot program with a duration of one year to evaluate the efficacy of microfiber filtration systems by installing and monitoring filters in ten state-owned laundry facilities [118]. When determining which strategies are the most effective overall for cutting down on the number of microfibers that are released, it is essential to take into account the mesh or pore size of the filters, in addition to the filtering and capture capacity of such filters. In addition, it has been found that employing laundry balls designed to gather fibers while washing can result in a 26% decrease in the number of fibers found in the effluent produced by the washing process [119]. Furthermore, it has been hypothesized that the use of washing bags to encase synthetic textiles during the laundering process would reduce the amount of microfibers released into the environment by collecting microfibers. Microfibers emissions can be reduced by adding a final filtering step to wastewater treatment facilities, such as sand filtration, Membrane Bioreactors (MBR) treatment, or pile fabric filtration. Stockholm, Sweden’s wastewater treatment facilities are now rebuilding a portion of their existing sludge system by constructing an MBR, which will make it the largest MBR facility in the world. Microfibers (particularly 50 μm) may be reduced by as much as 61% using granular activated carbon and other technologies often used to eliminate micropollutants in water treatment [120,121,122]. However, this type of technical progress may necessitate additional resources such as power, chemicals, and so on, as well as a considerable initial investment and continuing maintenance costs. To be clear, many regions of the world may not have the option of upgrading wastewater treatment facilities as described above owing to a lack of the requisite infrastructure. Improving sludge processes, such as pre-treatment to decrease microfiber concentration, is also beneficial. In recent years, clean-up technologies such as plastic collectors have been utilized to collect plastic garbage floating on the water’s surface and keep it from being moved downstream.

## 4. Machine Vision Can Control the Microfiber Releases

The textiles and garment production phase as well as the consumer phase is highly responsible for the microfiber releases. Before it could be controlled, this has to be measured in the right way to give a feedback loop to each sector (i.e., fiber to the fabric manufacturing stage and consumer phase) to have control measures and standards [123]. The postcondition of the apparel manufacturing process in fabric formation, dyeing, washing, garment processing, and denim washing. During the fiber-to-yarn conversion, fibers go through many stresses and strain processes in terms of mechanical treatment between the number of rollers to achieve the functions to open, paralyzation, and finally, its form of yarn. Spinnability includes several physical properties each influencing the ability of the fibers to be spun into yarn, for example, capable of taking a twist, a certain degree of friction, and providing additional surface resistance to abrasion. Those processes are enhancing the yarn quality and help to form the yarn formation, as a result, the fabric produced from those yarns makes better quality of the fabric. 

As already discussed, the microfibers are released from clothing and other textiles during the washing process. When these fabrics are washed, tiny fibers can break off and enter the wastewater stream, eventually ending up in the environment. Before we find a solution or make control measures for microfiber releases from garments in industrial/domestic washing, there are some questions that need to be answered. For example, in domestic washing, the additional filters might be helpful in lowering the amount of microfibers released into the environment; however, currently, there are more than 840 million washing machines in use across the world, and it would be very challenging to install filters in all of them at once [58]. At the moment, a number of manufacturers of washing machines are offering their products with filters that, according to their claims, dramatically cut down on the amount of microfiber emission [57]. However, it is necessary to make a chart such as the amount of cloth washed in the machine and the manual washing. According to the Kirsi Laitala work [124], Scandinavia holds the highest use of washing machine (i.e., 87%) and some of the nations such as India, Kenya, Vietnam, and Indonesia shows that only <40% of clothes are washed using washing machines. Particularly, the main method in rural areas of developing countries is hand washing and some of the developed countries such as the USA use dry cleaning more often. As a result, installing additional filters in washing machines could not be an effective strategy for reducing the total amount of microfibers released into the environment around the world.

**Figure 11:**
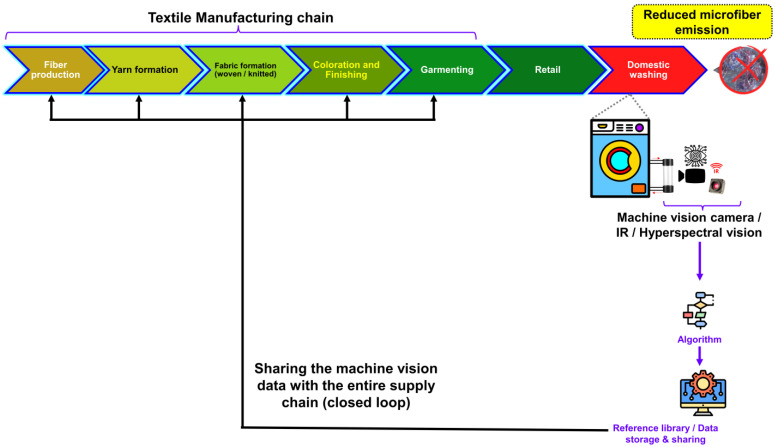
Scheme for the implementation of machine vision in the textile manufacturing chain.

Currently, there are a few methods to analyze microfibers emission, for example, DIN SPEC 4872 Test Method [125] and the AATCC test method 212-2021 [126]. However, these results are mostly carried out on the finished product (i.e., end of the supply chain) and the test results are not shared with the entire supply chain. Moreover, manual entry of these data is digitalized and then there will be human errors in the quantification of the data, additionally, it has repetition issues with respect to the results as well. Furthermore, another important point to keep in mind, is that the textile value chains were sourcing fibers from one country, and producing yarn, and fabric in another country, and the final product could be assembled in another country. During these long supply chains, the feedback of microfiber release is not transferred to the raw material processor where they can technically enhance the properties of the raw materials to improve the performance of the final products to reduce the microfiber releases and it is an open loop. Therefore, it is necessary to create a closed-loop system where feedback on test results needs to share in the complete supply chain to produce the lower microfiber release as a control measure in each process/sector of business. The closed-loop system for data sharing in the textile manufacturing chain is illustrated in Figure 11. This system is based on the data collected by machine vision while the textiles are being washed at home.

The development of technology and its possibilities, particularly in digital technologies, can be utilized to generate data that can be used to discover critical processes and threads, as well as ways to optimize supply chains. The creation of a control measure for the microfiber releases and the identification of the microfibers along with their length and width can both be accomplished through the use of machine vision, which is one of the best options conceptually. Those measures of cameras detected pictures can be converted into pixels and charts to identify when /where the microfiber release is higher in-term of different compositions. 

Machine vision technology, applied to the textile industry in the field of inspection, has the characteristics of being fast, real-time, accurate, and efficient. Machine vision inspection equipment replaces the human eye to complete surveillance, measurement, and judgment and has many application advantages such as non-contact, repeatable, reliability, high accuracy, continuity, high efficiency, and good flexibility [127]. The camera in this system collects information about the image of the fabric in real-time and sends it to the backend system. Following the completion of the deep learning process, the system is now able to determine whether or not the fabric depicted in the current image has a flaw, and if it does, it is able to determine whether or not a matching response operation should be performed for that flaw.

### 4.1. Image Analysis for Quantification of Microfibers/Microplastic Materials

Research on microplastics often makes use of image analysis as a quick method for determining the size [128,129,130,131] shape [132,133,134,135] and quantity [136,137,138,139] of individual plastic particles. To begin, the program that does image analysis provides the dimensions of the image in physical units. 

For fibers, the complete length (long axis) of the fiber can be traced using straight or segmented lines, which may be useful for determining volume given that the width of fibers is relatively symmetric [134]. Particle size was most commonly quantified as the Feret maximum [140]. In the event that this is not the case, a threshold image can be processed using a skeletonize function to narrow the fiber down to its centerline, which has a width of one pixel for the purpose of length calculation [135]. Because the fibers can be split into the mechanical agitation that occurs during the washing process and creates fibers, a bundle of fibers, fiber fragments, or fiber pellets, the microfibers that are released from domestic washing have the potential to be in a variety of diverse shapes. In point of fact, the fibers need to have an aspect ratio that is no less than 3:1 (length to diameter) [135].

The images of the microfibers that are present in the home washing are captured by cameras or other sensor devices under a variety of circumstances (i.e., detergent, water qualities, turbidity, foam). In this scenario, efficient microfiber identification might be improved by calibrating the camera power by the resolutions of the camera, pixel rate, and frame rate of the camera. Because it was tested in a turbidity environment, the number of frames it captures per second and the frame quality requirements are different when we utilize this for moving water rather than water that is kept in constant circumstances. Based on the above parameter, it is necessary to pick up the right sensor and pixel size (i.e., small pixel size gives data noise) of the sensor to determine the sensitivity. The sensitivity of the camera is determined by the amount of light that is available from the source that is being captured; however, sensitivity can be adjusted if the camera is being used in a controlled setting. In that case, a camera with a higher sensitivity will be necessary, and color sensitivity is yet another characteristic that needs to be re-examined.

To communicate with computers, these cameras must interface with computers that have strong rendering capabilities. These recorded, collected images are merged with image processing software, which is commonly used for an algorithm, image segmentation, and measurement feature extraction.

- Image segmentation: The image processing system identifies the objects to be measured or validated and eliminates the background or remaining contamination from the acquired image.
- Feature extraction: Once the objects have been authenticated and recognized, image processing software extracts the key measurements of the existence, diameter, and length of microfibers.

Measure the images in various ways, compute the fiber edges to determine the length, width, and so on, and provide a graphical representation of the quantity of water compared to the contamination level to the actual fiber level. Microfibers and other forms of microplastics are differentiated from non-synthetic microparticles by the use of visual classification as the traditional first step in the process [141]. Visual classification is unable to define the different forms of polymers, but it can indicate whether or not a particle is composed of plastic. Due to the dyed microfibers and the plastic-like look of some natural mineral materials, visual inspection has a significant possibility of producing false positive results. At this time, visual inspection by itself is not accepted as a method for classifying microfibers [142]. It is also necessary to feed the data in order to generate the findings in digital forms; however, it may cause variance in results and it differs from person to person, it will not be a good choice to transfer the data from this inspection share to the entire supply chain. 

#### Machine Learning Applications

Methods of segmentation are required in order to obtain statistically relevant information on the dimensions (i.e., length and diameter) of microfibers from the photographs that are produced by a machine vision camera. These segmentation methods must be able to separate the microfibers from the background as well as from one another objects. Although automated approaches for detecting and counting microfibers are well-established and widely used in medical image analysis (i.e., to detect cells), these methods are also suitable for the detection of microfibers. In the future, this method is promising for detecting and counting microfibers [143,144,145,146]. These approaches, on the other hand, frequently run into difficulties when attempting to classify irregular object patterns and background noise [147]. Additionally, some of these algorithms depend on hand-crafted feature selection or shape assumptions, which frequently limits their application to the detection of well-defined objects with a particular feature within the context of other objects that give rise to contrast in an image [145,146].

**Figure 12:**
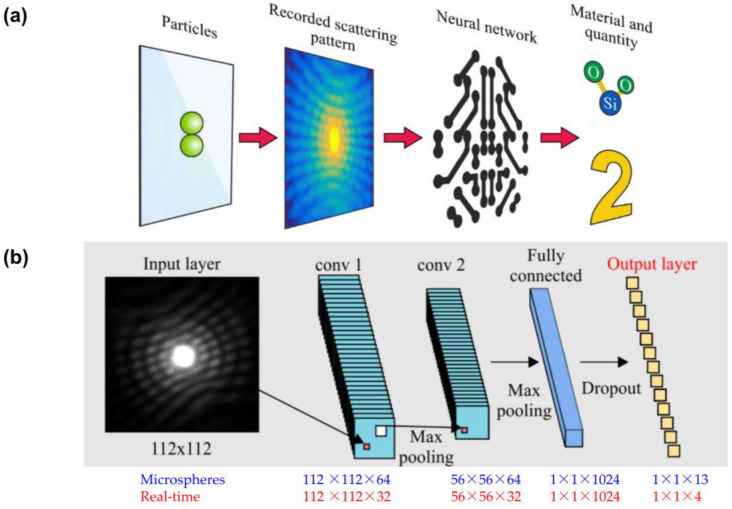
Proposed application of a NN to quantify an object directly from the scattering pattern. (**a**); Diagram of the NN used for the microspheres and real-time experiments (**b**). (Reused from [148], with Creative Commons 4.0 Permission).

James et al. [148] utilize machine learning to identify particles directly from scattering patterns of microplastics. Several breakthroughs in neural networks (NN) provide effective object categorization and diffraction imaging. When given the right training data, a NN can directly measure the substance and number of particles from a single scattering pattern, according to the authors’ proposed sensing approach (Figure 12a). The NN utilized for both the microspheres and the real-time tests is depicted in Figure 12b. Before feeding the NN the training data, the images were cropped to 112 by 112 pixels and normalized to 255. After the input layer, microspheres NN and real-time NN have 64 and 32 convolutions, respectively, with sizes of 112 by 112 and 56 by 56 for microspheres and 112 by 112 and 56 by 56 for real-time. Microspheres and real-time NN layers have kernel sizes of 3 and 15, respectively, with a pooling factor of 2 and stride of 2. The second layer output went to a fully linked 1024-neuron layer [148].

**Figure 13:**
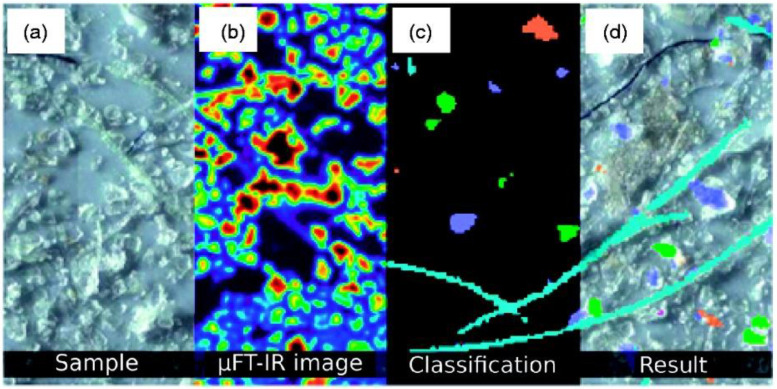
Classification of microplastics in µIR images using a machine learning approach: (**a**) optical image, (**b**) µIR image, (**c**) pixel-wise classification output of the machine learning model for the different polymer types (including PE, PP, PMMA, PAN, PS, and non-polymer particles), and (**d**) classification result overlaid on the optical image of the sample. (Reused from [158] with permission from The Royal Society of Chemistry Creative Commons Attribution 3.0).

Identifying microfibers and interpreting images can also be accomplished through the use of machine learning with trainable algorithms, in addition to the more conventional threshold-based methods. In recent years, with advancements in machine learning and particularly deep learning [149,150,151,152], deep convolutional neural networks (CNNs) have essentially replaced traditional algorithms in a variety of fields, ranging from medical image analysis with remarkable improvements in pattern and image recognition accuracy [153,154,155,156]. For instance, a CNN has been used in conjunction with photographs of microplastic microspheres and scattered laser light from microspheres in order to locate microspheres on a surface depending on the composition and shape of the microspheres. Later on, authors have been utilizing these concepts as a foundation to build a device to detect microplastic particles automatically [148]. Deep learning networks are able to attain performance on specialized tasks that is comparable to or even better than that of humans thanks to the capability of CNNs to automatically extract information relevant to a job from even the most complicated of data sets [157]. However, in order to train deep neural networks, companies will need large-scale data sets that include a significant amount of manual effort. For the textile supply chain, the acquisition of these data sets can be a time-consuming and financially burdensome process. Efforts to simplify the task, such as transferring the data across the organizations, can be made in order to ameliorate the current state of affairs. As can be seen in Figure 13, Hufnagl et al. [158] propose using a random forest approach to detect microplastics in µIR pictures, and they claim that their system successfully identifies multiple kinds of polymers using only a spectral map. Despite the promise of the method, there is still just a small amount of experimental evidence to back up this assertion (i.e., six polymer classification classes). As a result, the conclusion is that machine learning techniques are an adequate stepping stone toward achieving significant progress in fully automating the classification of plastic particles.

**Figure 14:**
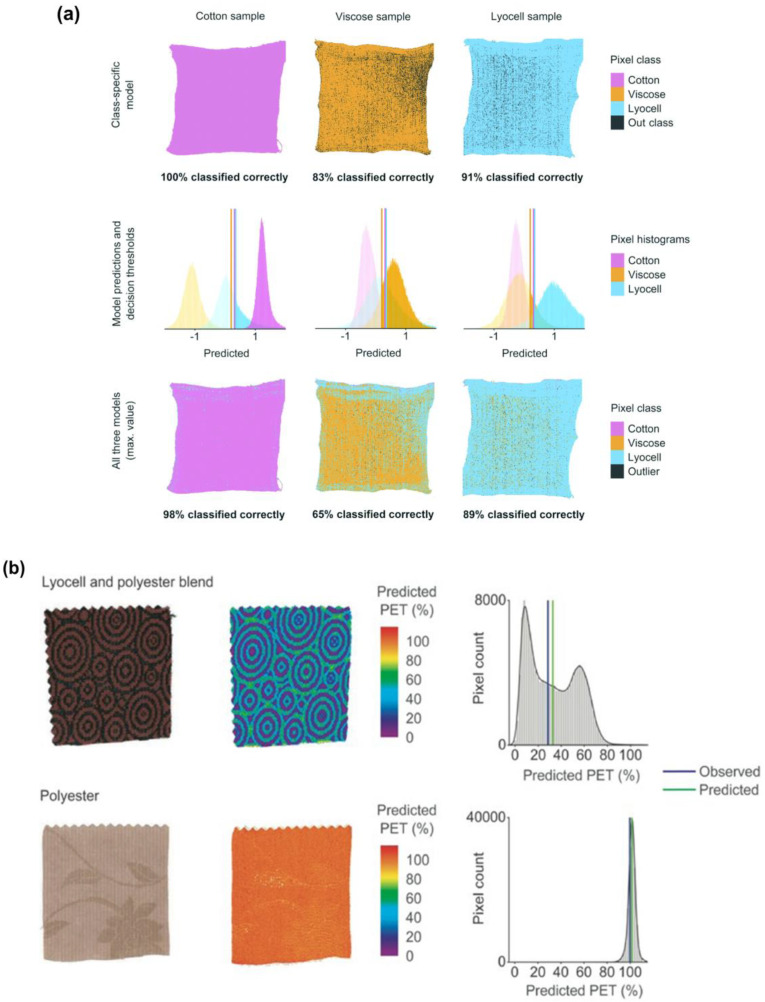
Classification results based on the pixel test set of cotton, viscose, and lyocell samples of known compositions (**a**) (Reused from [159], with permission from The Royal Society of Chemistry Creative Commons Attribution 3.0); Image predictions, their polyester contents based on image predictions (**b**) (Reused from [160], with permission from Elsevier Creative Commons Attribution license).

Makela et al. [159] used near-infrared (NIR) imaging spectroscopy to classify natural and regenerated cellulose fibers. Perhaps, this method has been utilized for sorting textile waste for recycling. However, this is a promising method to identify microfibers as well. The advantages of these techniques can differentiate the different cellulose fibers (i.e., cotton, viscose, lyocell) with their intrinsic viscosity (Figure 14a). The same team of researchers widened their investigation to identify the polyester lyocell blend fabrics and polyester fabric waste [160]. In Figure 14b, the predicted values for their polyester content in the images, and the pixel histograms visually represent the distributions of those expected values. 

### 4.2. Influence of Textile Functionalization on Image Capturing and Processing

Coloration (i.e., dyeing or printing) is a part of the textile value chain and it influences to identification and distinguish microfibers and their sources. In colorations, some dyes could penetrate the inside the fiber structure (i.e., reactive dyes [161,162], direct dyes [163], acid dyes [164], and natural dyes [165]) and some of them have physical adsorption on the fiber surface (i.e., pigments [166] and vat dyes [90,167]). Fibers such as silk and viscose rayon could be lustrous and they influence capturing the image and followed by the image processing. Some of the fibers have special dyes such as photo and thermochromic dyes have a strong influence on the identification [168,169,170,171]. Additionally, optical brighteners, optical brightening agents (OBAs), fluorescent brightening agents (FBAs), or fluorescent whitening agents (FWAs) are typically applied to white fabrics in order to improve their brightness and whiteness [172,173,174]. Additionally, FWAs are regularly added to home laundry detergents in order to improve the whiteness and brightness properties of garments after they have been laundered [175,176]. Since the various finishing techniques, such as flame retardancy [177,178] and water repellency [179,180], modify the material’s surface properties [181,182,183,184], they also have a significant impact on the image-capturing process. All of these factors have a significant impact on the image capture process, which is then followed by the quantitative analysis of microfibers.

#### 4.2.1. Reference Libraries and Matching Software

To validate the classification of microfibers, reference libraries make it possible to quickly obtain information regarding the forms, surface topologies, and colors of microfibers. Any spectral matching software relies heavily on its reference libraries to function properly [185]. There is currently no defined reference library that can be used for the identification of microfibers. However, there are some libraries (i.e., commercial libraries such as National Institute of Standards and Technology; KnowItAll, Bio-Rad; Polymer Spectral Libraries, Thermo Fischer Scientific), but these libraries deal with generalized domains such as microplastics and not for microfibers. Due to the high cost of these libraries, it is only available to those with more substantial budgets and it is possible for government or corporate organizations. Particles found in the environment frequently include additives (for example, dyes and plasticizers), and they have been subjected to weathering and/or degradation. Commercial libraries, on the other hand, tend to be made primarily of spectra from pure polymer reference materials. To this day, only a limited number of plastic libraries have been made available to the public, such as one for use with Raman spectroscopy [186] and one for IR [187]. Additionally, for visual classification, the reference images are often restricted in number and diversity and are difficult to gather from the literature. However, there have been several publications published under the microplastic domain that might be beneficial for the identification of microfibers [60,188,189,190,191,192].

#### 4.2.2. Library Matching Procedures

Although polymer libraries exist for the accurate classification of materials, issues still arise in the matching process. Microfibers from textile sources have in different forms and it contains different dyes, printing ingredients [193,194,195], finishing chemicals such as phosphorous (i.e., flame retardancy [196,197,198,199]), fluorocarbon and silane (i.e., water repellency) [170,200,201] and formaldehyde (i.e., crease recovery [202,203,204]) and with a variety of nanoparticles [205,206]. Because of this, the spectra that are typical of a single type of polymer might vary significantly from one another, depending on the particular dyes, printing, and finishing chemicals that are present in the fiber structure. Spectra collected from several spots on the same microfibers can look very different from one another. In addition, polymers obtained from environmental samples are frequently subjected to conditions such as photodegradation, thermal-degradation, and biodegradation, all of which have the potential to cause leaching or denaturing of chemical components, thereby changing the original polymer composition and, consequently, its chemical fingerprint [207]. 

#### 4.2.3. Data Sharing and Storage with the Supply Chain

In today’s hyper-digital world, the storing and sharing of data is of critical importance, and it has grown to be seen as an essential component of effective supply chain management practices. When considering safety and availability, data storage (i.e., server) is an important factor to take into account. It is possible to lose data as a result of insufficient data backup procedures, which become more complicated. For the reduction of microfiber emission, the quantified microfiber data should be openly disseminated among the various process of the textile value chain that enhance the performance of the entire textile supply chain to make it more sustainable. In the future, machine learning for the classification of microfibers, numerous methods and data fusion techniques, sophisticated machine learning approaches, and the easily accessible reference library will make machine learning quantification and data sharing for microfiber identifications more efficient. This will allow for more accurate identifications of microfibers.

## 5. Conclusions and Recommendations for Future Remarks

This review focuses on the sustainable way to reduce microfiber emissions during domestic washing. First, it reviewed the various textile properties and their influence on microfiber emissions, for example, there are many factors that influence the generation and release of microfibers in the textile materials including the type of fabric, fabric geometry (woven, knit, or nonwoven), yarn type (twist, evenness, hairiness, staple fiber, filament, and the number of fibers per cross-section), fabric processing history (i.e., spinning, knitting, or weaving, scouring, bleaching, dyeing, finishing. Aside from that, optimizing the washing conditions is a very important fact. For example, higher releases can be observed due to the oxidation and hydrolysis behavior of polyester-based textiles under alkaline conditions. Additionally, the higher washing temperature causes the fibers derived from cellulosic-based materials to degenerate after an extended period of washing. As temperatures rise, detergents cause surfaces to become wet, and fibers become more easily detached from yarns and fabric structures. In point of fact, the optimization of these textile properties is an essential aspect that is directly proportional to the reduction in the number of microfibers. Additionally, for optimization, these properties can be efficiently attained by the machine learning data that has been discussed in Section 4.

Modifying the surface levels of the fabrics is one approach that could be taken to address the problem of microfiber pollution. As a result, this article discussed the influence of mechanical and chemical finishes on microfiber emissions. Additionally, it identifies the research gaps that need to be filled in order to direct future research priorities in chemical finishing techniques. In mechanical finishing, shearing and brushing generate fuzz fibers on the surface of the fabric to provide comfort properties, despite the fact that shearing and brushing release a significant quantity of microfibers during domestic washing, other mechanical finishes such as singing and calendaring help control the microfiber generations. The coating of PLA, PBSA, and pectin on the fabric surface has shown significant results in the reduction of microfibers generations.

In last it discussed the potential and futuristic application of machine vision (i.e., quantification, data storage, and data sharing) to reduce the microfiber emission from domestic washing. Machine vision technology is already applied to the textile industry for quality inspection, as it is fast, real-time, accurate, and efficient. Recently, microplastics can be identified and quantified using the machine vision camera.

Nevertheless, there are a large number of aspects of the system as a whole that have room for development in order to become more effective, for instance, the efficient capture of the images can be controlled by the cameras under a variety of circumstances (i.e., detergent, water qualities, turbidity, foam). In this particular scenario, efficient microfiber identification might be improved by calibrating the camera power by the resolutions of the camera, pixel rate, and frame rate of the camera. Additionally, to communicate with computers, these cameras must interface with computers that have strong rendering capabilities. These recorded, collected images are merged with image processing software, which is commonly used for the algorithm, image segmentation, and measurement feature extraction. In conclusion, the machine vision information should be disseminated across the entire textile production chain so that the process parameters (i.e., the qualities of the output) can be optimized, hence contributing to a decrease in the number of microfiber generations.

Overall, to successful reduction of microfibers, required an easily available library, enhanced data storage, and data sharing facilities. In the future, machine learning for the classification of microfibers, numerous methods and data fusion techniques, sophisticated machine learning approaches, and the easily accessible reference library will make machine learning quantification and data sharing for microfiber identifications more efficient. This will allow for more accurate identifications of microfibers.

## Figures and Tables

**Table 1:** Types and properties of microplastics.

Microfibers	Chemical Formula	Polymeric Structures	Density (g/cm^3^)
PP	(C_3_H_6_)_n_	Semi-crystalline (isotactic, syndiotactic), Amorphous (atactic)	0.88–1.23
PET	(C_10_H_8_O_4_)_n_	Semi-crystalline	1.30–1.50
PA6 PA66	(C_6_H_11_NO)_n_ (C_12_H_22_N_2_O_2_)_n_	Semi-crystalline	1.12–1.14 1.13–1.38
PAN	(C_3_H_3_N)_n_	Semi-crystalline	1.1–1.18

**Table 2:** Influence of mechanical and chemical finishing on microfibers releases.

Finishing Techniques	Results	Ref
Finishing with chitosan	27% reduction of microfiber releases on polyester fabric. Poor durability and inconsistent results	[64]
Finishing with silicone and acrylic resin	Significant reduction of microfibers released from PET fabric. No durability measurement for both finishes.	[64]
Finishing with PLA	63.4% reduction of microfibers released from the treated PA fabric	[77]
Finishing with PBSA	76.8% reduction of microfibers released from the treated PA fabric	[77]
Finishing with pectin	90% reduction of microfibers released from the treated PA fabric	[74]
Mechanical finishing: singeing and calendaring	Significantly reduce the microfibers emission. Non-durable	[21]
shearing and brushing	Both finishing generates fuzz fibers on the surface of the fabric to provide comfort properties, as it discharges a significant quantity of microfibers during domestic washing.	[21]

## Data Availability

Not applicable.
